# Supplementary material for: At what cycle threshold level are dogs able to detect SARS-CoV-2 in humans?
Source: PLoS One. 2025 Jan 17;20(1):e0317213. doi: 10.1371/journal.pone.0317213 (PMC11741569; doi:10.1371/journal.pone.0317213)
Supplement: S1 File — (DOCX) [file pone.0317213.s001.docx]

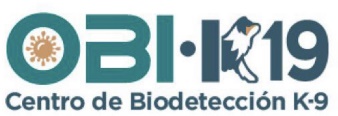

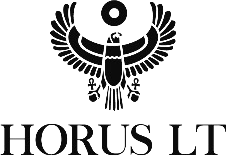
**Centro Canino de Detección de Enfermedad**


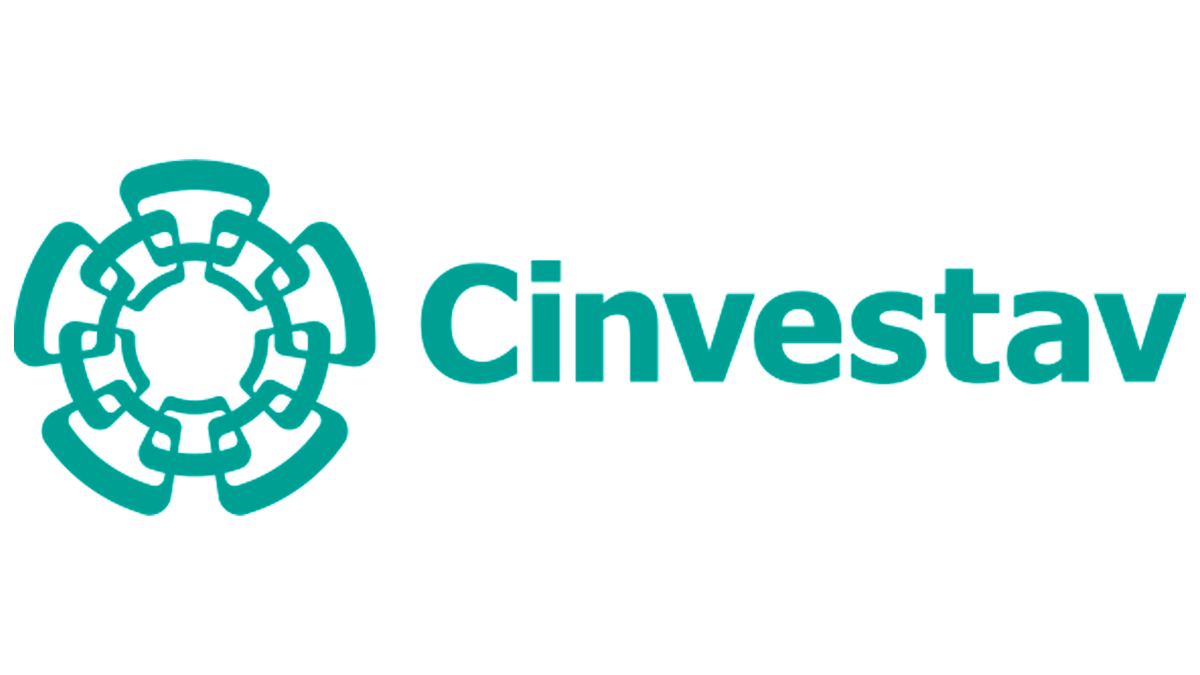
**OBI K-19**

CARTA DE CONSENTIMIENTO INFORMADO


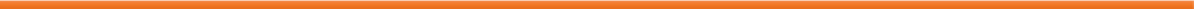


De acuerdo con las disposiciones contenidas en la Ley General de Salud, Titulo Quinto “Investigación para la Salud”, Capitulo Único, artículo 100, fracción IV; así como del Reglamento de la Ley General de Salud en Materia de Investigación para la Salud, Titulo Segundo “De los Aspectos Éticos de la Investigación en Seres Humanos” Capítulo I, Disposiciones Comunes, artículo 13 que señala que en toda investigación en la que el ser humano sea sujeto de estudio, deberán prevalecer el criterio del respeto a su dignidad y la protección de sus derechos y bienestar, artículos 14 fracción V, 20, 21 y 22 de dicho Reglamento; y, de conformidad con los principios éticos contenidos en la Declaración de Helsinki, se me ha explicado e informado que:

I. Se me ha explicado que posiblemente padezca de la infección viral SARS-CoV-2 (COVID-19) y que se me propone participar en el proyecto para probar la detección de ésta utilizando fluidos corporales como una posible alternativa a la detección por RT-PCR.

II. Se me ha informado que se tomarán muestras de sudor y saliva, las cuales son adicionales al estudio que requiere la prueba RT-PCR. Además, se me tomarán datos personales relacionados con mi edad, dieta, uso de medicamentos, etc.

III. Los resultados de este estudio ayudarán a determinar con mejor eficacia la presencia de SARS-CoV-2 (COVID-19) en mi caso y en el de otros pacientes.

IV. Se me ha asegurado que puedo preguntar hasta mi complacencia todo lo relacionado con el estudio y mi participación.

V. Autorizo la publicación de los resultados de mi estudio a condición de que en todo momento se mantendrá el secreto profesional y que no se publicará mi nombre o revelará mi identidad.

VI. Los investigadores se comprometen en proporcionarme información actualizada obtenida durante el estudio, aunque ésta pueda contradecir los resultados obtenidos en la prueba de RT-PCR.

VII. Los estudios de laboratorio (RT-PCR) y la detección mediante el uso de perros, serán cubiertos por el Centro Anticipa y el proyecto de investigación “OBI: Caninos contra COVID” respectivamente.

Con fecha____________________________, habiendo comprendido lo anterior y una vez que se me aclararon todas las dudas que surgieron con respecto a mi participación en el proyecto, yo_________________________________________ con número de paciente_____________________, acepto participar en el estudio titulado: “OBI: Caninos contra COVID”

Nombre y firma del paciente __________________________________________________________.

Nombre y firma del testigo ____________________________________________________________.
