# Supplementary material for: At what cycle threshold level are dogs able to detect SARS-CoV-2 in humans?
Source: PLoS One. 2025 Jan 17;20(1):e0317213. doi: 10.1371/journal.pone.0317213 (PMC11741569; doi:10.1371/journal.pone.0317213)
Supplement: S1 Table — The best model was that with the lowest value of the Akaike´s information criterion. (DOCX) [file pone.0317213.s002.docx]

S1 Table

| **Model** | **θ** |  | **Parameters** | | | | | **AICc** | Δ_AIC_ | **w%** |
| --- | --- | --- | --- | --- | --- | --- | --- | --- | --- | --- |
|  |  | **a** | **b** | **c** | **μ** | **σ** | **k** |  |  |  |
| **Ct N1** | | | | | | | | | | |
| Linear | 2 | 10.442  (8.961 – 11.922) | -0.240  (-0.283 – -0.196) |  |  |  |  | 965.991 | 52.280 | 4.43e-10 |
| Power | 2 | 30.14  (15.166 – 52.453) | -0.719  (-0.893 – -0.515) |  |  |  |  | 1031.929 | 118.219 | 2.13e-24 |
| Polynomial (2 degree) | 3 | 2.849  (-0.960 – 6.658) | 0.342  (0.068 – 0.616) | -0.010  (-0.014 – -0.005) |  |  |  | 950.533 | 36.822 | 1.00e-6 |
| Logarithmic | 2 | 20.555  (16.458 – 24.651) | -5.223  (-6.400 – -4.045) |  |  |  |  | 995.370 | 81.659 | 1.85e-16 |
| Gaussian | 3 |  |  |  | 23.005  (21.377 - 24.281) | 7.493  (6.541 - 8.711) | 6.872  (6.152 - 7.613) | 913.710 | 0.000 | 99.99 |
| **Ct N2** | | | | | | | | | | |
| Linear | 2 | 9.192  (7.805 – 10.580) | -0.196  (-0.236 – -0.156) |  |  |  |  | 886.887 | 69.447 | 8.29e-14 |
| Power | 2 | 10.202  (5.698 – 15.767) | -0.395  (-0.531 – 0.225) |  |  |  |  | 949.292 | 131.851 | 2.33e-27 |
| Polynomial  (2 degree) | 3 | -0.091  (-2.595 – 2.412) | 0.578  (0.390 – 0.765) | -0.013  (-0.017 – -0.010) |  |  |  | 829.382 | 11.941 | 0.254 |
| Logarithmic | 2 | 13.024  (9.660 – 16.387) | -3.026  (-3.994 – -2.058) |  |  |  |  | 929.451 | 112.010 | 4.74e-23 |
| Gaussian | 3 |  |  |  | 23.329  (21.584 – 24.789) | 8.449  (7.409 – 9.730) | 6.948  (6.243 – 7.667) | 817.440 | 0.00 | 99.74 |
| **Combined data (Ct N1 and Ct N2)** | | | | | | | | | | |
| Linear | 2 | 9.710  (8.705 – 10.715) | -0.215  (-0.244 – -0.186) |  |  |  |  | 1850.115 | 120.584 | 6.53e-25 |
| Power | 2 | 13.192  (8.818 – 18.333) | -0.475  (-0.576 – -0.356) |  |  |  |  | 1982.361 | 252.830 | 1.25e-53 |
| Polynomial  (2 degree) | 3 | 1.217  (-0.901 – 3.336) | 0.463  (0.308 – 0.618) | -0.011  (-0.014 – -0.009) |  |  |  | 1781.693 | 52.162 | 4.71e-10 |
| Logarithmic | 2 | 15.715  (13.135 – 18.296) | -3.817  (-4.560 – -3.075) |  |  |  |  | 1928.467 | 198.936 | 6.33e-42 |
| Gaussian | 3 |  |  |  | 23.011  (21.842 – 24.013) | 8.001  (7.263 – 8.873) | 6.866  (6.361 – 7.379) | 1729.531 | 0.000 | 99.99 |
