# Supplementary material for: At what cycle threshold level are dogs able to detect SARS-CoV-2 in humans?
Source: PLoS One. 2025 Jan 17;20(1):e0317213. doi: 10.1371/journal.pone.0317213 (PMC11741569; doi:10.1371/journal.pone.0317213)
Supplement: Attachment — Submitted filename: DOgCOVID2024.docx [file pone.0317213.s004.docx]

This is the review of the manuscript At what cycle threshold level are dogs able to detect SARS-CoV-2 in humans?

The COVID-19 pandemia was and is a health concern worldwide resulting in catastrophic results in many countries.

The authors performed the COVID-19 detection by trained dogs by using a gauze, and cycle threshold by RT-PCR against N1 and N2 SARS-CoV-2 gene. Some aspects should be addressed by the authors.

1. The abstract should be improved and then to impact the data obtained.
2. A large introduction is presented, and much information are outside for this.
3. There is a lack of information about the training procedure of the dogs. How was performed, time or period of training, samples used, pseudo-scent, what is the pseudo-scent, positive controls. Negative controls, confuse controls, etc.
4. Several repetitive information is readily available, this is confused to read.
5. The determination of copies is for sample or individual.
6. To many data in the manuscript, could be better a table to observe it.
7. Why the authors used number of virus copies, there is lack of information about the virus DNA detected by dogs. Do you have background about DNA detected by dogs?. A logic thought is the dog detects organic compounds that inert compounds (DNA). In this line, high viral load is directly related to high viral protein, is this correct?
8. The sensitivity and specificity values by each dog are different, obviously, different data should be obtained.
9. The data craving to compare rapid antigen detection with the dogs than the DNA detection. A real situation is, which is better detection for COVID-19 disease, dogs or rapid detection strip?
10. Regarding the clinical data of the patients included in the protocol, they were severe, mild COVID-19? This could discriminate among different clinical diagnoses.
11. The dogs are not machine which have loaded a program, they should be trained previously and then to challenge with the target. However, any dog doesn’t have the characteristic to work for specific work.
12. An important question is how much viral (antigen) protein is secreted (outside of the cell) and be detected by the dog. Of course, if you intended to work with dogs. Another is, if high viral load is directly proportional to high secreted proteins.
13. How reinforced for dogs were performed. The time involved to read all the samples. The read performed were in close room or in an open site.
14. Once obtained all the samples were immediately reads or were stored.
15. There is a lack of a conclusion.
16. The abstract should be improved and then to impact the data obtained.
17. The title of the manuscript is not related to data presented.
