# Supplementary material for: At what cycle threshold level are dogs able to detect SARS-CoV-2 in humans?
Source: PLoS One. 2025 Jan 17;20(1):e0317213. doi: 10.1371/journal.pone.0317213 (PMC11741569; doi:10.1371/journal.pone.0317213)
Supplement: Attachment — Submitted filename: Response to Reviewers.docx [file pone.0317213.s005.docx]

**Lilia C. Soler-Jiménez**

Aquatic Pathology Laboratory

CINVESTAV

July 18, 2024

Professor **Julian Ruiz-Saenz**

Academic Editor

PLOS ONE

Dear Dr. Julian Ruiz-Saenz

Attached you will find the manuscript entitled **“At what cycle threshold level are dogs able to detect SARS-CoV-2 in humans?”** by Victor Manuel Vidal-Martínez, Juan Manuel Mancilla-Tapia, Lilia Catherinne Soler-Jiménez, Iván Velázquez-Abunader, Matilde Jiménez-Coello, Antonio Ortega-Pacheco, David Hernández-Mena. All the editorial suggestions were considered in this new version of the manuscript. Below you will find detailed answers to each one of these suggestions.

**AUTHOR(S):** Victor Manuel Vidal-Martínez, Juan Manuel Mancilla-Tapia, Lilia Catherinne Soler-Jiménez, Iván Velázquez-Abunader, Matilde Jiménez-Coello, Antonio Ortega-Pacheco, David Hernández-Mena.

**Manuscript number:** PONE-D-23-33291

**TITLE:** At what cycle threshold level are dogs able to detect SARS-CoV-2 in humans?

We are truly grateful with the referees for their valuable comments. We included first the referee´s suggestion and/or question, followed by our answer in a paragraph starting with A/, an acronym that stands for “Answer”, and in blue color.

Looking forward to hearing from you soon

Yours sincerely

___________________________

Lilia Catherinne Soler-Jiménez, PhD.

Response to Reviewers

Reviewer #1: Comments to the Author

In the manuscript entitled “At what cycle threshold level are dogs able to detect SARS-CoV-2 in humans?”, the authors present the findings of high detection capacity of COVID-19 by dogs for Ct ranges between 16 and 29.

They conclude the possibility and utility for the rapid detection way of COVID-19 by trained animals in case of screening the crowd. This study provides valuable information, additional explanations should be required to improve the manuscript for the readers.

Major points

1. How many consecutive judgements by the dogs maintain the sensitivity and specificity? In the discussion section, the authors described “they (the dogs) can screen hundreds of people in a few hours”. but the testing period lasted for 12 weeks in this study. The discussion might be overestimated.

A/ Dogs can screen hundreds of people for SARS-CoV-2 in one hour, as we found during our contract with Purina Pet Care Company (<https://purina.com.mx/purina/purina-sociedad/obi-k19>). This method involved individuals placing their face masks in a hole for the dogs to sniff. The base for the development of this method was that during the Omicron variant outbreak in 2022, the canine training center OBI K9 was able to screen up to 200 workers in Hermosillo, Sonora with a single dog in just 25 minutes. It is important to mention that for a big Purina factory such as the one of Silao, Guanajuato, workers had a limited amount of time to enter the working area. So, our limit for screening 300 workers with the dogs was 25 minutes. During this experience, one of our dogs processed 200 workers and a second one 100 workers two times per day. Therefore, the dogs worked a total of two hours per day processing around 600 people daily.

2. The mechanism of detecting the SARS-CoV-2 by the dogs smelling the sweats seemed not to be clear. Did the severity of COVID-19, the patients’ immune status or the kinds of the variants of SARS-CoV-2 influence? If the possibility was evaluated, the data should be added.

A/ For this study, it was considered crucial to use samples from highly symptomatic patients, particularly those experiencing loss of taste and smell. We were certain about whether the patient was infected with SARS-CoV-2 based on the results of the RT-qPCR. However, factors such as the patient's immunological status or virus variant, were not included in the study due to financial restrictions. The importation into Mexico of molecular biology kits for the detection of variants is just too expensive.

3. Did the Ct values of sweats and nasopharyngeal samples in one person show the same?

A/ The Ct values were obtained only for the RT-qPCR method, based on nasopharyngeal samples. The sweat samples were only sniffed by the dogs. However, we obtained very good correlations for the Ct values of the N1 and N2 regions of the nucleocapsid of the virus. This means that in most cases the Ct value and the reading obtained by the dog were showing the same.

Minor points:

1. In figure 1D, the range of Ct values between 60 and 100 might not be needed.

A/We appreciate the comment of the reviewer. However, we decided to keep the Ct range of the figure, because it allows us to show that there is significant variability between the N1 and N2 regions.

2. Figure legends found in the results section. It should be moved to the appropriate section.

A/ We moved the figure legends as suggested.

3. How often the training is required to maintain the ability of detecting the virus?

A/ Dogs are trained 3 to 5 days a week after 6 weeks resting period.

**Reviewer #2:**

The authors performed the COVID-19 detection by trained dogs by using a gauze, and cycle threshold by RT-PCR against N1 and N2 SARS-CoV-2 gene. Some aspects should be addressed by the authors.

1. The abstract should be improved and then to impact the data obtained.

A/ We appreciate the reviewer´s comment. The abstract was modified as suggested. A clearer explanation was provided about the variability in the detection of dogs and its relationship with the viral load and the methods used in the study. In addition, the use of language was improved by being more concise and direct to improve the readability and understanding of the text.

2. A large introduction is presented, and much information are outside for this.

A/ We modified the introduction as suggested. The changes made in the text included the elimination of unnecessary repetition of phrases, simplification of some expressions, some phrases were reformulated to avoid redundancy and the reorganization of some parts, all to improve the fluidity, coherence and clarity of the text.

3. There is a lack of information about the training procedure of the dogs. How was performed, time or period of training, samples used, pseudo-scent, what is the pseudo-scent, positive controls. Negative controls, confuse controls, etc.

A/ The training procedure of the dogs was described in detail in Mancilla et al. (2022). These authors used two methods for training the dogs. In the first method, exercises and scenarios are detailed to reinforce odor recognition and improve the dogs' attention. The importance of selecting an appropriate reward to keep the dog focused and balanced, avoiding distractions, is emphasized. It also highlights the need to create a positive work environment for the dog to associate detection with a pleasant experience. Additionally, it is mentioned that the training process varies slightly between the methods, with the first method more based on the use of a toy (Kong) as a reward, while in the second method, scent is introduced using food as a reward from the beginning of the training procedure. The transition to using the Kong alone occurs after the dogs demonstrate understanding of the scent.

The information about pseudo-scent and positive/negative control was detail in the materials and methods section of the manuscript (lines 113-120):

“The pseudo-scent used for dog training was designed by the American company Bio Detection K9 (BDK9), which is a protein that mimics the scent of COVID-19. According to BDK9, this pseudo-scent closely resembles the odor associated with COVID-19. BDK9 has employed this pseudo-scent to train dogs to detect COVID-19 and screen individuals at large gatherings. The positive controls consist of sweat or saliva samples obtained from patients diagnosed with COVID-19, confirmed through RT-PCR or Antigen testing. The negative controls consist of sweat or saliva samples from individuals confirmed to be negative for COVID-19 through RT-PCR testing”.

4. Several repetitive information is readily available, this is confused to read.

A/ We have tried to improve the readability of the manuscript, removing all the information considered repetitive.

5. The determination of copies is for sample or individual.

A/ The determination of the copies was for individual.

6. To many data in the manuscript, could be better a table to observe it.

A/ We used three tables and one figure to summarize all the information in the manuscript. Probably, the Materials and Methods section is long, but the reason to present it in this way was to provide enough detail to other researchers to be able to repeat our experiments.

7. Why the authors used number of virus copies, there is lack of information about the virus DNA detected by dogs. Do you have background about DNA detected by dogs?. A logic thought is the dog detects organic compounds that inert compounds (DNA). In this line, high viral load is directly related to high viral protein, is this correct?

A/ We used the number of copies of the virus as an indirect measure of the viral load present in individuals infected with SARS-CoV-2. While dogs detect volatile organic compounds (VOCs), it has not been specifically identified whether they are detecting virus DNA or metabolic products associated with viral infection.

As for the background on the DNA detected by the dogs, there is no direct evidence to support this claim. Studies on the ability of dogs to detect disease, including the detection of SARS-CoV-2, generally focus on the ability of dogs to identify unique odors associated with the disease, such as VOCs produced by the infected organism.

It is true that a high viral load is directly related to greater production of viral proteins in the infected organism. However, dogs could detect VOCs released during the viral replication process or as a result of the body's immune response to infection. The exact relationship between viral load and organic compounds detected by dogs has not yet been definitively established and could be the subject of additional research.

8. The sensitivity and specificity values by each dog are different, obviously, different data should be obtained.

A/ Yes, and this because the different capacity of the dogs to detect the VOCs produced by the infected organism. Indeed, we realized that some dogs are much better for the VOC detection than other. In this sense, we recommend reproducing these top dogs to maintain their detection capacity and to accelerate the training period.

9. The data craving to compare rapid antigen detection with the dogs than the DNA detection. A real situation is, which is better detection for COVID-19 disease, dogs or rapid detection strip?

A/ Although the dogs do not meet the sensitivity and specificity standards set by the OMS, compared to commercially available rapid antigen detection tests, in many cases, the dogs' results were better. Their ability to perform large-scale testing in short periods of time makes them a valuable tool in the fight against the spread of COVID-19, especially in areas where RT-qPCR and rapid antigen tests are limited or expensive.

10. Regarding the clinical data of the patients included in the protocol, they were severe, mild COVID-19? This could discriminate among different clinical diagnoses.

A/ For this study, it was considered crucial to use samples from highly symptomatic patients, particularly those experiencing loss of taste and smell. However, the severity of the illness of the patient or his/her clinical condition were not considered in the data analysis.

11. The dogs are not machine which have loaded a program, they should be trained previously and then to challenge with the target. However, any dog doesn’t have the characteristic to work for specific work.

A/ Absolutely, not all dogs are suitable for detecting COVID-19. Some lack the necessary drive, concentration, ability, or interest to become effective working dogs. Interestingly, even among breeds known for their work capabilities, such as German Shepherds and Belgian Shepherds, not all individuals possess the traits required to excel as scent detection dogs.

Moreover, it's crucial to emphasize that being a biodetection dog for COVID-19 requires a higher level of concentration compared to dogs trained for detecting narcotics or explosives. This is because biodetection dogs need to discriminate among a greater number of volatile organic compounds (VOCs). Therefore, not every dog trained for scent detection can fulfill the requirements of a proficient biodetection dog. As said above, we recommend reproducing top dogs to maintain their detection capacity and to accelerate the training period.

12. An important question is how much viral (antigen) protein is secreted (outside of the cell) and be detected by the dog. Of course, if you intended to work with dogs. Another is, if high viral load is directly proportional to high secreted proteins.

A/ We appreciate the reviewer´s comment. To determine how much viral protein is secreted by the cells and how much can be detected by dogs, specific studies would be required. These studies could involve measuring the amount of viral protein present in biological samples (such as blood, saliva or urine) from infected patients (Jones et al. 2019), as well as evaluating the sensitivity of dogs to detect different levels of viral protein. Now, in relation to the viral load and protein secretion, this can vary depending on the virus and the state of the infection. In general, a higher viral load might be expected to be associated with increased secretion of viral proteins, as active viral replication generally leads to increased production of viral components, including proteins. However, this relationship is not always direct or proportional. Factors such as the host immune response, the phase of infection, and the ability of the virus to evade detection can influence the amount of viral proteins secreted relative to the viral load (Patel and Jain 2017). However, to answer these questions in detail, specific studies would be required using viral infection models and techniques for measuring viral load and viral proteins, as well as evaluating the performance of dogs in detecting different levels of viral proteins, which were not the objective of this study and therefore were not carried out.

13. How reinforced for dogs were performed. The time involved to read all the samples. The read performed were in close room or in an open site.

A/ The dogs in the study were reinforced in three different ways: through food rewards, using toys or Kongs, or petting them. The study was conducted in a laboratory, specifically in a room environment. The comprehensive evaluation or statistical examination involving the 11 dogs spanned a period of 4 weeks. Each dog sniffed an average of 24 samples daily, completing about 6 rounds throughout the day. These details were described in Mancilla et al. (2022).

14. Once obtained all the samples were immediately reads or were stored.

A/ Our goal was to analyze the samples immediately after collection. However, in many cases this was not possible, some samples were stored for 2 to 3 weeks before analysis, in constant freezing. It is worth noting that for other studies we have stored samples for up to a year, and even after such long storage, the dogs have been able to detect the volatile organic compounds (VOCs) associated with the samples.

15. There is a lack of a conclusion.

A/ The reviewer is correct. Therefore, the conclusions were included at the end of the discussion section.

“In conclusion, the ability of the dogs to detect the virus is related to the threshold cycle (Ct) values of the RT-qPCR test in a specific range, in which the dogs demonstrated a better detection capacity. This relationship suggests that dogs may be effective in discriminating between positive and negative cases of SARS-CoV-2 in real-life settings, especially in resource-limited settings. Likewise, the results of this study show that, although there was an improvement in the detection capacity of the second generation of dogs trained to detect SARS-CoV-2, there are still challenges in terms of sensitivity and specificity. Although six of the eleven dogs demonstrated a sensitivity between 67% and 87%, and all dogs showed a specificity between 87% and 92%, some did not reach sensitivity values considered optimal. However, compared to commercially available rapid antigen detection tests, in some cases, the dogs' results exceeded the results of such tests. Although the dogs did not meet the sensitivity and specificity standards set by the WHO, their ability to perform large-scale screening in short periods of time makes them a valuable tool in the fight against the spread of COVID-19, especially in areas where RT-qPCR and rapid antigen testing are limited or expensive. However, the importance of continuing research and training to further improve the ability of dogs to detect SARS-CoV-2 and other viral respiratory infections is noted”.

16. The abstract should be improved and then to impact the data obtained.

A/ The abstract was modified as suggested. A clearer explanation was provided about the variability in the detection of dogs and its relationship with the viral load and the methods used in the study. In addition, the use of language was improved by being more concise and direct to improve the readability and understanding of the text.

17. The title of the manuscript is not related to data presented.

A/ Our apologies, but we consider that the title of the manuscript is related to the data presented. This is because we found that the number of dog´s detections followed a gaussian model within a specific range of Ct values. Out of this range the number of dogs detections dropped dramatically. Then clearly, there was a clear relationship between the success of dog´s detections and the Ct values of the RT-qPCR. Consequently, we consider that the title of the manuscript is adequate.

**References**

Jones J, Qureshi Z, Pattenden L (2019). Detection and quantification of viral proteins. In Viral Pathogenesis (pp. 139-157). Humana, New York, NY.

Mancilla-Tapia JM, Lozano-Esparza V, Orduña A, et al. (2022) Dogs Detecting COVID-19 from sweat and saliva of positive People: A Field Experience in Mexico. Front Med (Lausanne) 9: 837053.

Patel JR, Jain RK (2017) Quantification of viral antigens. In Clinical Virology Manual (pp. 111-124). Springer, Cham.
