# Supplementary material for: At what cycle threshold level are dogs able to detect SARS-CoV-2 in humans?
Source: PLoS One. 2025 Jan 17;20(1):e0317213. doi: 10.1371/journal.pone.0317213 (PMC11741569; doi:10.1371/journal.pone.0317213)
Supplement: Attachment — Submitted filename: Response to Reviewers.docx [file pone.0317213.s006.docx]

**Lilia C. Soler-Jiménez**

Aquatic Pathology Laboratory

CINVESTAV

September 26, 2024

Professor **Julian Ruiz-Saenz**

Academic Editor

PLOS ONE

Dear Dr. Julian Ruiz-Saenz

Attached you will find the manuscript entitled **“At what cycle threshold level are dogs able to detect SARS-CoV-2 in humans?”** by Victor Manuel Vidal-Martínez, Juan Manuel Mancilla-Tapia, Lilia Catherinne Soler-Jiménez, Iván Velázquez-Abunader, Matilde Jiménez-Coello, Antonio Ortega-Pacheco, David Hernández-Mena. All the editorial suggestions were considered in this new version of the manuscript. Below you will find detailed answers to each one of these suggestions.

**AUTHOR(S):** Victor Manuel Vidal-Martínez, Juan Manuel Mancilla-Tapia, Lilia Catherinne Soler-Jiménez, Iván Velázquez-Abunader, Matilde Jiménez-Coello, Antonio Ortega-Pacheco, David Hernández-Mena.

**Manuscript number:** PONE-D-23-33291

**TITLE:** At what cycle threshold level are dogs able to detect SARS-CoV-2 in humans?

We are truly grateful with the referees for their valuable comments. We included first the referee´s suggestion and/or question, followed by our answer in a paragraph starting with A/, an acronym that stands for “Answer”, and in blue color.

Looking forward to hearing from you soon

Yours sincerely

___________________________

 Lilia Catherinne Soler-Jiménez, PhD.

Response to Reviewers

Reviewer #2: The SARS-CoV-2 virus by using trained dogs is still a huge challenge.

The authors should be addressing very carefully several aspects about their manuscript.

1.It is not clear what second generation of dogs mean? Are they F1?, Etc.

A/ We appreciate the reviewer´s comment. The second generation refers to a new group of dogs that were trained based on previous experience with the first group of SARS-CoV-2 detection dogs, anticipating deficiencies to be improved in this second group of dogs. Lines 97-102 in the revised version of the manuscript clarify the point about the first generation of dogs.

2.Each dog has specific characteristics, and they will have variability.

A/ Indeed, we recognize that each dog has unique characteristics and that there is variability in their ability to detect volatile organic compounds (VOCs) associated with infection. To control for variability in dogs’ olfactory abilities to detect volatile organic compounds (VOCs) associated with SARS-CoV-2, we assessed several aspects: 1. Each dog was individually tested for sensitivity and specificity in detecting VOCs. The results showed that some dogs had better olfactory abilities than others. This variability was measured by systematic testing, where the sensitivity of some dogs reached up to 87%, while others had marginal performance. 2. To mitigate individual differences, multiple dogs were used in sample detection, allowing animals to be rotated to avoid fatigue and ensure continuous performance. This rotation also allowed for comparing the results of different dogs on the same samples, which helped to identify and mitigate variability. 3. The Gaussian model was used to analyse the dogs’ detection, adjusting performance to different levels of viral load (measured by Ct values in RT-qPCR tests). This model helped to identify optimal detection ranges for dogs, within which they performed best, and allowed for variability outside of those ranges to be controlled. In summary, variability between dogs was managed through individual assessments, dog rotations, and the use of statistical models to optimize and adjust detection ranges.

3.Did you expect to obtain near of 50% (6/11) of dogs with better results? It is a little bit high percentage.

A/ Yes, it is a high percentage of dogs expected to improve their performances, but based on the increased time of impregnation of the gauzes with axillary sweat and saliva, and the additional training time received by the dogs, we considered reasonable to expect such an improvement.

4.The authors define some clinical characteristics of the patients, fever, pain, etc, could the authors to confirm mild or severe COVID-19 symptoms for the patients?

A/ Even when we were able to obtain the clinical characteristics of each patient, the only reliable way to establish whether that patient was positive or negative to SARS-CoV-2 was by using RT-qPCR tests.

5.They should discuss if the dog gender (male vs female) has any relation with the detection?

A/ In this study did not explicitly explore the influence of gender on the detection abilities of dogs, considering that in several studies on canine olfaction have not found conclusive evidence that gender significantly affects a dog's ability to detect volatile organic compounds (VOCs) (Johnen et al. 2017, Ellis et al. (2021). For example, Johnen et al. (2017) examines different factors that influence scent detection in dogs, including breed, training, and individual characteristics, without finding a significant gender effect. Likewise, Ellis et al. (2021) discusses variables that influence olfactory performance in dogs and concludes that gender is not a significant predictor of performance in detection tasks. Both male and female dogs have been successfully trained for similar detection tasks, suggesting that individual traits such as temperament, training, and motivation may play a more substantial role in their performance.

6.In the manuscript should be stated if the dogs are detecting the viral genome or some volatile compounds associated to the infection. This is confused. Moreover, in some sections looks like the virus particle detection than genome.

A/ As suggested by the reviewer, in the first paragraph of the introduction section was clarified that the dogs are detecting volatile compounds associated with the infection (Lines 68-71).

“It should be noted that detection dogs trained to identify viral diseases do not detect the viral genome directly. Instead, they recognize changes in VOCs, which are byproducts of altered metabolism due to infection (Grandjean et al. 2020, Jendrny et al. 2021)”.

7.Do the anecdotical experience is relevant?

A/ In this case, we consider that personal experiences are relevant because they provide qualitative and quantitative examples of the concepts raised. For example, in the specific case of the experience with Purina Pet Care in Mexico, this anecdote shows a practical case where detector dogs were used to identify possible cases of SARS-CoV-2 in a large population (14,000 workers), supporting the hypothesis about the accuracy and reliability of the method, by not finding positive cases during a period of three months. In addition, it provided us with evidence of the successful use of dogs in a real, uncontrolled environment, which gives credibility to the use of this approach outside of clinical or experimental contexts. On the other hand, it demonstrates the feasibility of implementing mass detection programs using dogs in work or social environments, demonstrating their ability to track large numbers of people continuously.

8.They mention that dogs possess a significant advantage, because they can screen hundreds of people in a few hours on a daily basis, compared with the more expensive and logistically complex application of RADs. Is it accepted that a dog can screen hundreds of people in a few hours on daily basis?. This is a serious mistake, because a dog has only minutes to work not hours.

A/ The reviewer is correct, stating that dogs can "work for hours" is a mistake. This was an error in the wording of the text. We are clear that, while it is true that detection dogs have the capacity to perform sniff tests in large volumes, they also have practical limitations. One of these is working time; dogs can work efficiently for short periods (usually between 20-30 minutes) before needing breaks. If it is a long day, dogs should rotate with other dogs to avoid fatigue. It is for this reason, that in this as in others studies we have conducted, we ensure that several trained dogs are available for rotation, ensuring their efficiency and well-being is maintained."

9.The authors are claiming that. Although the dogs did not meet the sensitivity and specificity standards set by the WHO, their ability to perform large-scale screening in short periods of time makes them a valuable tool in the fight against the spread of COVID-19, especially in areas where RT-qPCR and rapid antigen testing are limited or expensive. This mean the probability will be the answer, this should be well discussed.

A/ We appreciate the reviewer's comment and agree that it is essential to further discuss the likelihood that dogs can be an effective tool in mass screening. For this reason, we include the following paragraph in the discussion section:

Lines 553-563. “While the dogs' specificities do not yet meet the WHO's (2021) standards (≥97-100%), their ability to perform rapid detections on a large scale remains a valuable aspect. However, it is necessary recognize that for dogs to be considered a viable complementary tool, their success rate needs to be further analyzed in different scenarios, considering the prevalence of the disease in the population. Although dogs may not be a direct substitute for molecular testing in terms of specificity, their strategic use in specific contexts, could have a positive impact on outbreak containment, provided that limitations are properly considered.”.

10.At present, COVID-19 is and will be another frequent viral disease, the discussion about more emergent viral diseases by RNA virus is very superficial. This a huge challenge.

A/ We appreciate the reviewer's comment on the need for further discussion of other emerging viral diseases, especially those caused by RNA viruses. RNA viruses, as seen with SARS-CoV-2, represent a constant threat due to their high mutability and potential to generate new variants and pandemics. We have therefore expanded this discussion to better reflect the impact of emerging RNA viruses and the potential of dogs as alternative detection tools:

Lines 634-644: Finally, while our study primarily focused on the detection of SARS-CoV-2, it is important to acknowledge the broader context of emerging viral diseases, particularly those caused by RNA viruses. The high mutation rates of RNA viruses pose a continuous threat, as evidenced by recent pandemics like COVID-19 [35]. RNA viruses such as Zika, Ebola, and Influenza have demonstrated their potential to cause widespread outbreaks (Musso et al. 2016; Fauci et al. 2012). The ability to rapidly detect such viruses is critical in managing future pandemics. Although the dogs in this study did not meet the specificity standards set by the WHO, their capacity to perform large-scale screening in short periods of time makes them a valuable tool in the fight against emerging RNA viral diseases, especially in areas where testing resources are limited or expensive. Expanding the application of detection dogs to other RNA viruses could enhance global preparedness for future outbreaks.

11.The authors should discuss more about clinical and biological validation of the data.

A/ We used all of the available clinical variables and response variables, with all the proper standardizations in a canonical correlation analysis. There was a significant statistical association between the epidemiological data of the patients, and the number of dogs detections and the Ct values in canonical correlation analysis for both the first (F = 0.1; p = 0.0002, n = 367) and all canonical axes (F= 1.7; p = 0.0002). However, the amount of explained variance was only 6.03%. Consequently, we considered this association irrelevant. We added the information for the analysis in both the material and methods section (lines 301-306) and in the results section (453-458).

12.It is a suggestion, to avoid first person in the manuscript.

A/ The corresponding modifications were made to the text, according to your suggestion.

13.To avoid using “can, might”, is much better “could”.

A/ Thank you very much for your comment. We have taken your suggestion into account, considering the meaning and purpose of the sentence. Consequently, in many cases, we used "could" as you suggested, but in others, we decided to keep "can." For example:

- In the sentence, "Our hypothesis was that dogs should have a specific Ct range at which they could detect people infected with SARS-CoV-2," the use of "could" is appropriate. "Could" here suggests a capability in a hypothetical context or within the framework of a supposition, which aligns with the context of a hypothesis. If referring to an ability that you expect dogs to have under certain conditions, "could" is the correct choice.
- In the sentence, "Dogs can detect volatile organic compounds (VOCs) in sweat," the option "can" is the most appropriate if you are referring to a general and proven ability of dogs. "Can" indicates a present ability that dogs have habitually or inherently.
- In the sentence, "Thus, with sensitivity values between 67 and 87%, the results obtained by the dogs fall well within this range and can be considered an important tool for discriminating between positive and negative people with SARS-CoV-2, especially in resource-limited settings where RADs or RT-qPCR tests may not be readily available," the use of "can" is appropriate. "Can" indicates that the results obtained by the dogs have the capacity to be considered an important tool for discriminating between positive and negative individuals in resource-limited settings, which is a valid and suitable assertion for the context of the sentence.

14.Thus, the abstract, discussion and conclusion should be improved.

A/ The abstract, discussion and conclusion were improved, according with your suggestions.

15.Finally, to review the tables look like confused, and separate the data when the reports were done for viral antigen or genome detection.

A/ Our apologies but we do not see where the confusion in the Tables. Table 1 was about the characteristics of the dogs, Table 2 was about the sensitivity and specificity of the dogs for detecting SARS-CoV-2, and Table 3 was about the number of dogs detecting SARS-CoV-2 and the mean Ct values at which the dogs were able to detect positive and negative cases. Also, all our analyses were based on the use of Ct values from RT-qPCR tests. Consequently, we did not use viral antigen tests. What we did in the Discussion section was to compare the sensitivity and specificity results of our dogs with those of the Rapid Antigen Detection immunoassays (RADs) from the literature.

**Reference**

Ellis, J. L., Thomason, J., & Peña, A. S. (2021). "The effect of age, breed, and sex on olfactory behavior in domestic dogs (Canis lupus familiaris)." Journal of Veterinary Behavior, 42, 55-61.

Fauci, A. S., & Morens, D. M. (2012). The perpetual challenge of infectious diseases. New England Journal of Medicine, 366(5), 454-461.

Grandjean D, Sarkis R, Lecoq-Julien C, et al. Detection dogs as a help in the detection of COVID-19 Can the dog alert on COVID-19 positive persons by sniffing axillary sweat samples? Proof-of-concept study. PLoS ONE 2020; 15(12): e0243122.

Jendrny P, Twele F, Meller S, Osterhaus ADME, Schalke E, Volk HA. Canine olfactory detection and its relevance to medical detection. BMC Infect Dis 2021; 21(1): 838.

Johnen, D., Heuwieser, W., & Fischer-Tenhagen, C. (2017). "Canine scent detection—Fact or fiction?" Applied Animal Behaviour Science, 189, 1-10.

Musso, D., Gubler, D. J., & Tang, H. (2016). Zika virus infection. New England Journal of Medicine, 374(16), 1552-1563.
