# Supplementary material for: At what cycle threshold level are dogs able to detect SARS-CoV-2 in humans?
Source: PLoS One. 2025 Jan 17;20(1):e0317213. doi: 10.1371/journal.pone.0317213 (PMC11741569; doi:10.1371/journal.pone.0317213)
Supplement: Attachment — Submitted filename: Response to Reviewers.docx [file pone.0317213.s007.docx]

**Lilia Catherinne Soler Jiménez**

Aquatic Pathology Laboratory

CINVESTAV

December 18, 2024

Professor **Julian Ruiz-Saenz**

Academic Editor

PLOS ONE

Dear Dr. Julian Ruiz-Saenz

Attached you will find the manuscript entitled **“At what cycle threshold level are dogs able to detect SARS-CoV-2 in humans?”** by Victor Manuel Vidal-Martínez, Juan Manuel Mancilla-Tapia, Lilia Catherinne Soler-Jiménez, Iván Velázquez-Abunader, Matilde Jiménez-Coello, Antonio Ortega-Pacheco, David Hernández-Mena. All the editorial suggestions were considered in this new version of the manuscript. Below you will find detailed answers to each one of these suggestions.

**AUTHOR(S):** Victor Manuel Vidal-Martínez, Juan Manuel Mancilla-Tapia, Lilia Catherinne Soler-Jiménez, Iván Velázquez-Abunader, Matilde Jiménez-Coello, Antonio Ortega-Pacheco, David Hernández-Mena.

**Manuscript number:** PONE-D-23-33291

**TITLE:** At what cycle threshold level are dogs able to detect SARS-CoV-2 in humans?

We are truly grateful with the referees for their valuable comments. We included first the referee´s suggestion and/or question, followed by our answer in a paragraph starting with A/, an acronym that stands for “Answer”, and in blue color.

Looking forward to hearing from you soon

Yours sincerely

**Lilia Catherinne Soler Jiménez**

Response to Reviewers

Journal Requirements:

Please review your reference list to ensure that it is complete and correct. If you have cited papers that have been retracted, please include the rationale for doing so in the manuscript text, or remove these references and replace them with relevant current references. Any changes to the reference list should be mentioned in the rebuttal letter that accompanies your revised manuscript. If you need to cite a retracted article, indicate the article’s retracted status in the References list and also include a citation and full reference for the retraction notice.


Reviewer #2: This review is dedicated to authors Vidal-Martínez et al about, At what cycle threshold level are dogs able to detect SARS-CoV-2 in humans?

Several aspects should be addressed and discussed in depth to improve the proper manuscript.

1. In the first generation 4 out 6 (66%) dogs marked when using 1 minute. For the second generation 5 out 11 (aprox. 45%) marked.

A/ Thank you for your observation. To clarify, the performance of the second generation (5 out of 11, approximately 45%) reflects that these five dogs exhibited marginal results, as their 95% confidence intervals (CI) overlapped the randomness region (50%). This indicates that their results were statistically unreliable and likely influenced by chance. In contrast, the remaining six dogs in the second generation demonstrated statistically reliable performance, with 95% CI not overlapping the randomness region. This variability within the second generation highlights the challenges in achieving consistent results across a larger cohort and underscores the need to evaluate individual reliability. For the first generation, with fewer dogs (4 out of 6 marked, 66%), the majority displayed reliable performance, as their 95% CI did not overlap the randomness region. These differences between generations are discussed in the manuscript, and we have added further clarification to avoid misinterpretation.

2. Is confused if all participants were RT-PCR tested. How many patients with Antigen positive were detected by the dogs? What about for the RT-PCR? And then to compare both data.
A/ Positive samples were obtained mainly from two sources: Biostudio and the Progreso Health center in Progreso. These samples had a previous diagnosis, performed by RT-PCR in the case of Biostudio and by antigen testing at the Progreso Health center. Additionally, we performed RT-PCR tests on all samples, both negative and positive. Therefore, all samples evaluated by the dogs were confirmed by RT-PCR to verify their infection status. This procedure was carried out in order to establish a solid reference standard to validate the results obtained by the dogs.

The methodology section in the manuscript was modified to clarify this point to avoid confusion (Line 128 to 135): “The positive controls consisted of sweat samples obtained from patients diagnosed with SARS-CoV-2, the negative controls consisted of sweat samples from individuals confirmed to be negative for SARS-CoV-2, all samples confirmed through RT-PCR testing. In the case of positive samples obtained from the Progreso Health Center and the Biostudio Private Laboratory, these had already been previously diagnosed with an antigen test or RT-PCR, respectively. However, all samples (positive and negative) were subsequently tested and confirmed in this study by RT-PCR for consistency and standardization”

3. The authors should show, how was the detection limit and the number of viral copies calculus?
A/ Thank you for your comment. Below, we provide a detailed explanation of how the limit of detection (LoD) and the number of viral copies were calculated in this study. This information is also detailed in the methodology session:

Calculation of the Limit of Detection (LoD): A standard positive control (2019-nCoV PC67102, Norgen Biotek Corp, Ontario, Canada) with a known concentration of 200,000 copies/μL was used. Serial dilutions of this positive control were performed to cover a wide range of viral copy concentrations. Each dilution was analyzed using RT-qPCR, and the resulting Ct values were used to construct a standard curve. The relationship between the logarithmic concentrations (log10) of the dilutions and their respective Ct values was employed to establish a linear regression equation that defines the detection limit range. Calculation of the Number of Viral Copies: Once the regression equation (slope and y-intercept) was established from the standard curve, it was applied to estimate the number of viral copies in the patient samples. The Ct values obtained for the N1 and N2 regions of the SARS-CoV-2 N gene were processed using the regression equation, allowing the relative number of viral copies in each sample to be calculated. All calculations were performed using InfoStat software, ensuring precision and consistency.

This methodology enabled us to determine both the detection limit and the number of viral copies in the processed samples, providing a solid basis for the quantitative analyses conducted in the study. If you require additional details or specific figures related to the LoD, we can include them in the manuscript.

4. According to the symptoms, the dogs are for mild COVID-19, is this correct?
A/ Thank you for your question. The dogs in this study evaluted samples from individuals with varying symptoms, including mild, moderate, and potentially severe cases of COVID-19. Specifically, the samples obtained from the Progreso Health Center were exclusively from symptomatic individuals, as testing at this facility was limited to patients presenting symptoms such as fever, headache, cold-like symptoms, or diarrhea. This suggests that the samples included a range of clinical severities, likely covering mild, moderate, and possibly severe cases. Additionally, the study included samples from asymptomatic individuals collected at the Biostudio Laboratory. This laboratory provided a broader range of samples, as testing was also conducted on individuals without symptoms who sought screening for other reasons. As a result, the dogs were exposed to a diverse set of samples, encompassing symptomatic (mild to severe) and asymptomatic cases.

5. There is lack information about how the pseudo-scent was used, good or not for detection, could you please give some more information about the pseudo-scent?

A/ Thank you for your observation. The pseudo-scent used in our study was developed by the American company Bio Detection K9 (BDK9). This compound is a protein designed to mimic the specific odor of SARS-CoV-2, enabling the training of detection dogs without the need to handle infectious samples. BDK9 has employed this pseudo-scent in training programs for dogs tasked with detecting SARS-CoV-2 and screening individuals in large gatherings. Although specific information on the efficacy of BDK9's pseudo-scent is not widely available in the scientific literature, previous studies have demonstrated that dogs can be trained with synthetic compounds that emulate the volatile organic compounds (VOCs) associated with various diseases, including COVID-19 (Mancilla-Tapia et al. 2022).

In our study, the BDK9 pseudo-scent was used as an initial training tool for the dogs. Subsequently, the dogs were exposed to real sweat samples from individuals confirmed positive or negative for SARS-CoV-2 through RT-PCR testing. This mixed approach allowed us to evaluate the dogs' ability to generalize detection from the pseudo-scent to real biological samples. The results indicated that the dogs were able to accurately identify positive samples, suggesting that the BDK9 pseudo-scent is an effective tool for initial training. However, we acknowledge the need for additional studies to evaluate the comparative efficacy of the pseudo-scent versus real biological samples and its applicability in different detection scenarios.

5. The sex dog influenced for the detection? Most of the dogs are male.

A/ In this study did not explicitly explore the influence of gender on the detection abilities of dogs, considering that in several studies on canine olfaction have not found conclusive evidence that gender significantly affects a dog's ability to detect volatile organic compounds (VOCs) (Johnen et al. 2017, Ellis et al. (2021). For example, Johnen et al. (2017) examines different factors that influence scent detection in dogs, including breed, training, and individual characteristics, without finding a significant gender effect. Likewise, Ellis et al. (2021) discusses variables that influence olfactory performance in dogs and concludes that gender is not a significant predictor of performance in detection tasks. Both male and female dogs have been successfully trained for similar detection tasks, suggesting that individual traits such as temperament, training, and motivation may play a more substantial role in their performance.

6. Who dogs were coccidiomycosis affected? How many rounds they recovered the detection
A/ Thank you for your question. During the study, three dogs (Ziggy, Rufus, and Lucky) were diagnosed with coccidioidomycosis, likely due to their exposure to endemic environments. The affected dogs were treated with itraconazole, following standard antifungal protocols. After completing the treatment, all three dogs fully recovered and successfully returned to their detection tasks. They were given approximately one month off to resume training and regain their performance level. No further health complications were observed during the remainder of the study.

7. please to define 378 or 379 patients

A/ Thanks for the comment. This was a typo, which has already been corrected throughout the document. There were 379 samples analyzed.

**Major comments**

The introduction section is too long

A/ We modified the introduction as suggested. The changes made in the text included the elimination of unnecessary repetition of phrases, simplification of some expressions, some phrases were reformulated to avoid redundancy and the reorganization of some parts, all to improve the fluidity, coherence and clarity of the text.

The discussion section looks like results.

A/ Thank you for your comment. We have carefully reviewed the discussion section and removed any elements that presented results. The revised discussion now focuses on interpreting the findings, contextualizing them within the existing literature, and highlighting their implications, limitations, and relevance.

The authors should discuss the results. Not necessarily all the second-generation dogs should be better than the first one.

A/ Thank you for your observation. We discuss this point based on the hypothesis that the second generation of trained dogs would perform better than the first was only partially supported. While six of the 11 second-generation dogs showed sensitivity values between 67% and 87%, five of them achieved sensitivity above random detection levels (>50%). Comparatively, the first generation had six dogs with sensitivity values ranging from 58% to 80%. Specificity also improved in the second generation, with values between 87% and 92%, compared to 69% to 75% in the first generation. However, only six second-generation dogs consistently outperformed their predecessors in sensitivity. These results highlight among variability individual dogs and suggest that factors such as training or genetics may play a role. We have clarified this in the discussion section to ensure a balanced interpretation of the findings (Lines 416 to 426).

The abstract could be improved highlighting the results.

A/ We appreciate the reviewer's comment. The abstract was modified as suggested. We highlight the most relevant results of our research.

A better title could be considered.

A/ Our apologies, but we consider that the title of the manuscript is appropriate. This is because we found that the number of dog´s detections followed a gaussian model within a specific range of Ct values. Out of this range the number of dogs detections dropped dramatically. Then clearly, there was a clear relationship between the success of dog´s detections and the Ct values of the RT-qPCR. Consequently, we consider that the title of the manuscript is adequate.

It should be noted that there is a lack of information about the biochemical molecules involved, in other words, if there is a correlation (as you mention) between detection by dogs and N1/N2 viral proteins present in the patients.

A/ Thank you for your comment. Theoretically, dogs were trained to detect volatile organic compounds (VOCs) associated with SARS-CoV-2 infection, which are emitted by infected individuals. These VOCs reflect metabolic changes induced by the virus. However, the direct biochemical correlation between VOCs with the presence of viral proteins such as N1 and N2 was not specifically analyzed in this study. The correlation mentioned in the manuscript refers to the association between the dogs' detection accuracy and the Ct values obtained from RT-qPCR testing, which quantify the viral RNA concentration targeting the N1/N2 regions. This relationship suggests that higher viral loads (lower Ct values) may produce more pronounced metabolic changes and VOC emissions, enhancing detection by the dogs. We acknowledge the need for further biochemical analyses to identify the specific VOCs linked to SARS-CoV-2 and their potential connection to viral proteins. This limitation has been clarified in the revised discussion section to avoid overinterpreting the findings.

Could you please explain the difference between 1 minute vs 5- or 10-minutes gauze exposed?

A/ Thank you for your comment. For the first-generation evaluation of dogs trained to detect SARS-CoV-2, they were exposed to gauze soaked in sweat or saliva for one minute. Based on this experience, we considered it reasonable that longer exposure to the soaked gauze would improve the dogs' efficiency in virus detection. Therefore, for this study, dogs were exposed to gauze soaked in axillary sweat for 5 minutes. The text was modified to improve the explanation of this point and avoid confusion.

On the other hand, the 10 minutes refers to the time the patient kept the gauze in his armpit to soak it with sweat.

Line 89 to 98: For this first generation, four of the six dogs detected positive samples from patients with SARS-CoV-2, with sensitivity and specificity values significantly different from those obtained randomly in the field. These dogs were exposed to gauze impregnated with sweat or saliva from patients from Hermosillo for 1 min. We considered it promising that the performance of the dogs would improve by exposing them for a longer time to gauze impregnated with sweat or saliva from people with SARS-CoV-2. Therefore, in the field work for this study, the dogs were exposed for 5 minutes to gauze impregnated with axillary sweat from 379 people in Mérida, Yucatán, Mexico (90 positive samples and 289 negative samples) to evaluate the effectiveness of the SARS-CoV-2 biodetection training received by the dogs.

**Reference**

Mancilla-Tapia JM, Lozano-Esparza V, Orduña A, et al. Dogs Detecting COVID-19 from sweat and saliva of positive People: A Field Experience in Mexico. *Front Med (Lausanne)* 2022; **9**: 837053.
